# Cytohesin-3 is required for full insulin receptor signaling and controls body weight via lipid excretion

**DOI:** 10.1038/s41598-019-40231-3

**Published:** 2019-03-05

**Authors:** Bettina Jux, Dominic Gosejacob, Felix Tolksdorf, Christa Mandel, Michael Rieck, Angrit Namislo, Alexander Pfeifer, Waldemar Kolanus

**Affiliations:** 1 Department of Molecular Immune and Cell Biology, Life and Medical Sciences (LIMES) Institute, University of Bonn, Bonn, Germany; 2 Institute of Pharmacology and Toxicology, University of Bonn, Bonn, Germany

## Abstract

Insulin plays a central role in regulating metabolic homeostasis and guanine-nucleotide exchange factors of the cytohesin family have been suggested to be involved in insulin signal transduction. The *Drosophila* homolog of cytohesin-3, *steppke*, has been shown to be essential for insulin signaling during larval development. However, genetic evidence for the functional importance of cytohesin-3 in mammals is missing. We therefore analyzed the consequences of genetic cytohesin-3-deficiency on insulin signaling and function in young and aged mice, using normal chow or high-fat diet (HFD). Insulin-receptor dependent signaling events are significantly reduced in liver and adipose tissue of young cytohesin-3-deficient mice after insulin-injection, although blood glucose levels and other metabolic parameters remain normal in these animals. Interestingly, however, cytohesin-3-deficient mice showed a reduced age- and HFD-induced weight gain with a significant reduction of body fat compared to wild-type littermates. Furthermore, cytohesin-3-deficient mice on HFD displayed no alterations in energy expenditure, but had an increased lipid excretion instead, as well as a reduced expression of genes essential for bile acid synthesis. Our findings show for the first time that an intact cyth3 locus is required for full insulin signaling in mammals and might constitute a novel therapeutic target for weight reduction.

## Introduction

Metabolic diseases like type 2 diabetes are widespread in western countries and are rapidly arising in developing countries. The development of type 2 diabetes depends on the combination of several risk factors like genetic causes, lifestyle (e.g. overweight), and age. The prevalence of diabetes is estimated to rise from 171 million in 2000 to 366 million in 2030^1^ due to higher life expectancy increasing both the elderly and the obese population. Therefore, the basis of insulin signaling and the regulation of nutrient metabolism under different conditions i.e. starvation, food intake, high-fat diet, and aging are under intense investigation.

Recently, members of the cytohesin protein family, a group of guanine-nucleotide exchange factors (GEF) for ADP-ribosylation factor GTPases, have been implicated in modulating insulin receptor (IR)-signaling via the PI3K/AKT pathway^2,3^. The protein structure of cytohesins consists of an coiled-coil domain for protein-protein interaction, a PH domain that enables recruitment to the plasma membrane by binding to the phosphatidylinositol-phosphates PtdIns(4,5)P_2_ and/or PtdIns(3,4,5)P_3_ and a sec7 domain that harbors the GEF-activity^4^. Stimulation of 3T3-L1 adipocytes with insulin induces a translocation of cythohesin-3 (cyth3) to the plasma membrane^5^ and insulin-stimulated actin rearrangement depends on cyth3 expression and function^6^. Cytohesin function can be inhibited by using the pan-cytohesin inhibitor secinH3 which binds to the sec7 domain and thereby blocks the GEF activity^2^. Treatment of 3T3-L1 adipocytes with secinH3 inhibits the insulin-stimulated recycling of glucose transporter 4^7^ and treatment of mice with secinH3 leads to hepatic insulin resistance^2^. Moreover, knock-down of cyth3 in the human hepatocarcinoma cell line HepG2 was sufficient to abrogate the insulin response^2^. The knock-down or inhibition of the cytohesin orthologue *steppke* in *Drosophila melanogaster* indicated an essential role for cytohesin proteins in insulin signaling^8^. All studies conducted so far are based on the use of antibodies, siRNA approaches, or the application of secinH3. Furthermore, there is a complete lack of data about the *in vivo* role of cytohesin family members for metabolism by using knockout mice.

This study provides the first metabolic analysis of cyth3 full knockout mice (cyth3^−/−^). Phenotypic alterations of these animals were analyzed with respect to insulin signaling and the regulation of nutrient metabolism after metabolic changes like starvation and re-feeding, after insulin injection, during high-fat diet, and aging. Cyth3^−/−^ mice do not develop an overt diabetic phenotype but do show significantly reduced IR activity in liver and adipose tissue. Under aging conditions or on a high-fat diet, cyth3^−/−^ mice exhibit reduced weight gain accompanied by decreased accumulation of body fat due to increased fat excretion. In conclusion we found cyth3 to play an important role in insulin signaling and body fat regulation *in vivo*.

## Results

### Loss of Cytohesin-3 does not cause a diabetic phenotype in healthy, young mice on a chow diet

In this study, we analyzed the role of the cytohesin-3 (cyth3) gene in metabolism and IR-signaling in the mammalian system in liver and adipose tissue under different conditions by using a cyth3-deficient (cyth3^−/−^) mouse line. Western blot analysis revealed that cyth3 is expressed in all metabolically relevant organs i.e. liver, muscle, adipose tissues, and pancreas (Supplementary Fig. S1a). Cyth3^−/−^ mice are vital, fertile, and healthy. Total body weight (Supplementary Fig. S1b) and organ weights of young adult (7–12 weeks old) male and female wt and cyth3^−/−^ mice were comparable (Supplementary Fig. S1c). Furthermore, cyth3-deficient mice show no overt signs of diabetes (Supplementary Fig. S1d–i).

### Cyth3 expression is necessary for full IR-signaling in liver

**Figure 1:**
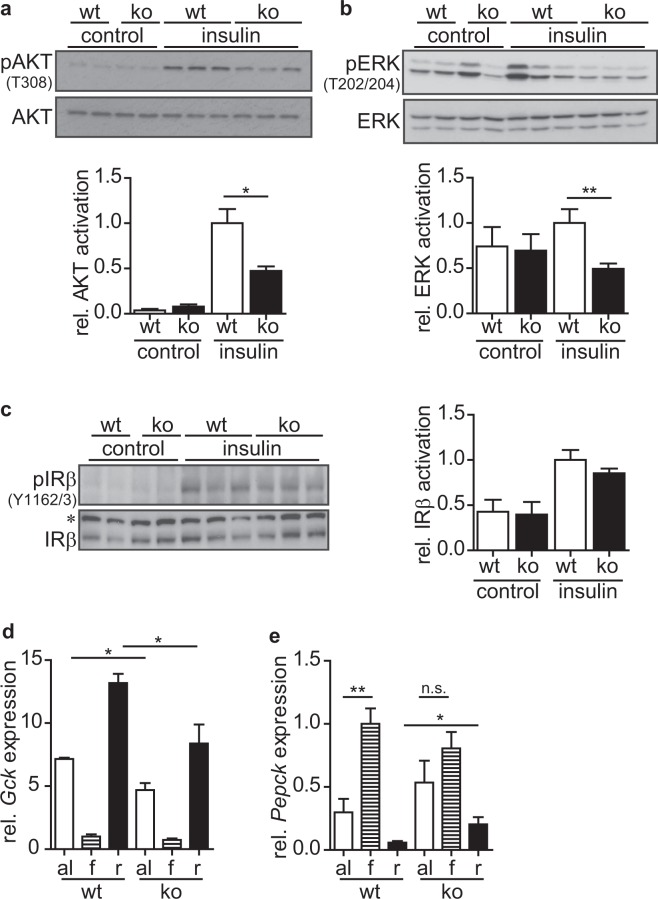
Cytohesin-3 is required for full IR-activation in the liver. 10 minutes after i.p. injection of non-fasted male and female wt (white bars) and cyth3^−/−^ (ko, black bars) mice with insulin (0.75 U/kg of body weight) livers were removed and the activation of AKT (**a**) ERK (**b**) and the insulin receptor (IR) (**c**) was measured as a ratio of phosphorylated to total protein levels. Representative Western blot analyses are shown in which each lane represents an individual mouse (*Unspecific band). The activation was normalized to insulin-stimulated wt mice (A, B: n = 6 control mice, 2 male and 4 female mice; 9 insulin-injected mice, 3 male and 6 female mice; C: n = 3 control mice, 2 male and 1 female mice; 6 insulin-injected mice, 3 male and 3 female mice). Gene expression of glucokinase (*Gck*) (**d**) and *Pepck* (**e**) in livers from male and female wt and cyth3^−/−^ (ko) mice was analyzed by PCR with food ad libitum (al, white bars, n = 6), after a starvation period for 12 hours (f, hatched bars, n = 8) and after re-feeding for four hours (r, black bars, n = 8) (n = 6–8, 2–4 male and 4 female mice). The expression was normalized to *Hprt* and calculated in comparison to fasted wt livers, which were set to 1. The results are given in means + SEM (*p < 0.05; **p < 0.01; n.s. = not significant).

We tested whether cyth3 is necessary for direct IR-signaling based on previous studies demonstrating a role for cytohesins in modulating IR-signaling^2,8^. We injected insulin intraperitoneally (i.p.) and assessed IR-activation by phosphorylation of the IR-downstream targets, AKT and ERK1/2, and the IR by western blot. Livers from cyth3^−/−^ mice showed a significantly lower activation of AKT (Fig. 1a) and ERK1/2 (Fig. 1b) after insulin injection compared to wt mice. Phosphorylation of the IR was comparable in wt and cyth3^−/−^ livers (Fig. 1c) indicating that cyth3 is located downstream of the IR and upstream of AKT and ERK1/2.

Organisms are permanently challenged by uptake of nutrients or by periods of starvation. To clarify the physiological consequences of the reduced IR-signaling in livers of cyth3^−/−^ mice, we analyzed gene expression in the liver with normal chow ad libitum, 12 hours after starvation, and after subsequent re-feeding for four hours. As expected, expression of glucokinase (*Gck*) was strongly repressed during fasting and up-regulated during re-feeding (Fig. 1d). *Gck* expression was significantly reduced in livers from re-fed cyth3^−/−^ mice compared to wt mice. In food ad libitum samples, *Gck* expression in cyth3^−/−^ livers was significantly lower than in wt mice. IR-signaling also led to a differential expression of *Pepck*. *Pepck* was strongly induced by starvation and repressed by re-feeding (Fig. 1e). In contrast to wt mice, repression by re-feeding was significantly reduced in cyth3^−/−^ mice. Furthermore, gene induction following starvation in cyth3^−/−^ mice was not found to be significant because of an already high expression of *Pepck* with food ad libitum. These results corroborate an important role of cyth3 in liver following starvation and re-feeding but also under standard feeding conditions. Cytohesin-proteins are activators of Arf-GTPases which regulate membrane trafficking and actin dynamics^9^. Insulin-induced actin rearrangements have been shown to be dependent on cyth3 and Arf6^6^. Therefore, we asked whether IR-internalization is affected after cyth3-knock-down and thereby regulates signaling using HepG2 cells. AKT activation after insulin stimulation was reduced by 50% in HepG2 cells after cyth3-knock-down (Supplementary Fig. S2a), comparable to the effect on liver after i.p. injection of insulin in cyth3-deficient mice. Surface expression of the IR after insulin stimulation for 10–30 minutes (determined by flow cytometry) showed a severely reduced internalization in HepG2 cells after cyth3-knockdown (Supplementary Fig. S2b) and could therefore account for the reduced insulin signaling observed in cyth3-deficient liver.

Taken together, these results highlight an important role of cyth-3 for full activation of IR-signaling in liver possibly due to regulation of IR-internalization.

### Cytohesin-3 expression is indispensable for IR-signaling in adipose tissue

**Figure 2:**
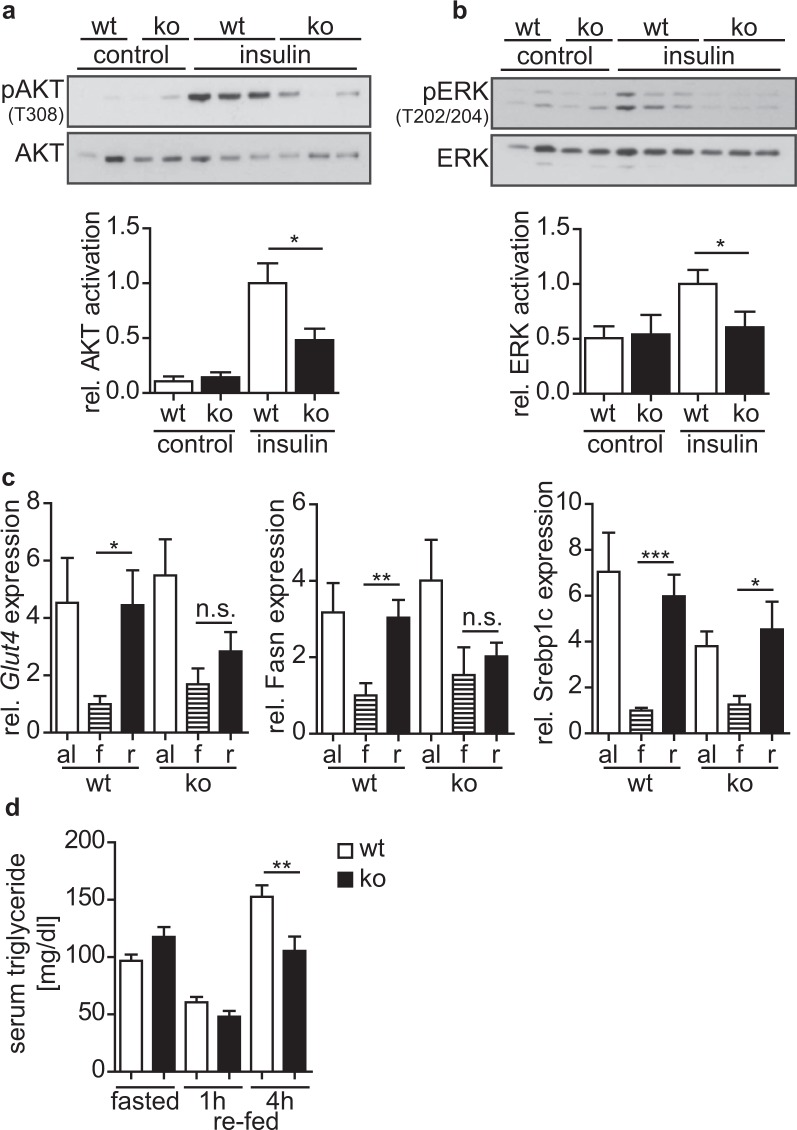
Cytohesin-3 expression is indispensable for IR-signaling in inguinal subcutaneous white adipose tissue (WATi). 10 minutes after i.p. injection of non-fasted male and female wt (white bars) and cyth3^−/−^ (ko, black bars) mice with insulin WATi was removed and immediately stored in liquid nitrogen. The activation of AKT (**a**) and ERK (**b**) was measured as a ratio of phosphorylated to total protein levels. Representative western blot analyses are shown where each lane represents an individual mouse. The activation in insulin-stimulated wt WATi was set to 1 for calculation (n = 6 control mice, 2 male and 4 female mice; 8 insulin-injected mice, 2 male and 6 female mice). Gene expression of *Glut4;* Fatty acid synthase (*Fasn*), and sterol regulatory element binding transcription factor 1c (*Srebp1c*) was analyzed by PCR in WATi from male and female wt and cyth3^−/−^ (ko) mice with food ad libitum (al, white bars, n = 6), after a starvation period for 12 hours (f, hatched bars, n = 7) and after re-feeding for four hours (r, black bars, n = 8) (n = 6–8, 2–4 male and 4 female mice) (**c**). The expression was normalized to *Hprt* and calculated in comparison to the expression in fasted wt WATi, which was set to 1. (**d**) Serum triglyceride levels were measured by ELISA from starved (n = 8) and subsequently re-fed wt (white bars) and cyth3^−/−^ (ko, black bars) mice after one (n = 6) and four (n = 8) hours (n = 6–8, 3–4 male and 3–4 female mice). The results are given in means + SEM (*p < 0.05; **p < 0.01; ***p < 0.001; n.s. = not significant).

The *in vivo* role of cyth3 in adipose tissues is not yet known. Based on our finding that cyth3 is directly involved in IR-signaling in the liver, we analyzed the response of the subcutaneous inguinal white adipose tissue (WATi) to metabolic changes by PCR and western blot. Insulin injection induced a strong activation of AKT and ERK1/2 in wt mice as detected by their phosphorylation (Fig. 2a,b) which was diminished by 40–50% in cyth3^−/−^ mice demonstrating the essential role of cyth3 for IR-activation in WATi.

We furthermore analyzed the gene expression of key genes implicated in glucose flux toward de novo lipogenesis (*Glut4;* Fatty acid synthase - *Fasn*, and sterol regulatory element binding transcription factor 1c - *Srebp1c*) in WATi of wt and cyth3^−/−^ mice after starvation and re-feeding (Fig. 2c). All genes were repressed during starvation and significantly up-regulated after re-feeding in wt mice while in cyth3^−/−^ mice, neither *Glut4* nor *Fasn* were significantly up-regulated after re-feeding. *Srebp1c* expression was by trend lower in cyth3^−/−^ mice compared to wt mice after re-feeding. In accordance with this impaired gene induction, we detected a significantly reduced generation of triglycerides in knockout mice after re-feeding in comparison to wt mice (Fig. 2d). Expression of genes essential to lipolysis was not significantly regulated under the tested conditions (data not shown).

In summary cyth3 is essential for lipogenesis and full IR-signaling in adipose tissue.

### Cytohesin-3 deficient mice are glucose tolerant and insulin tolerant

**Figure 3:**
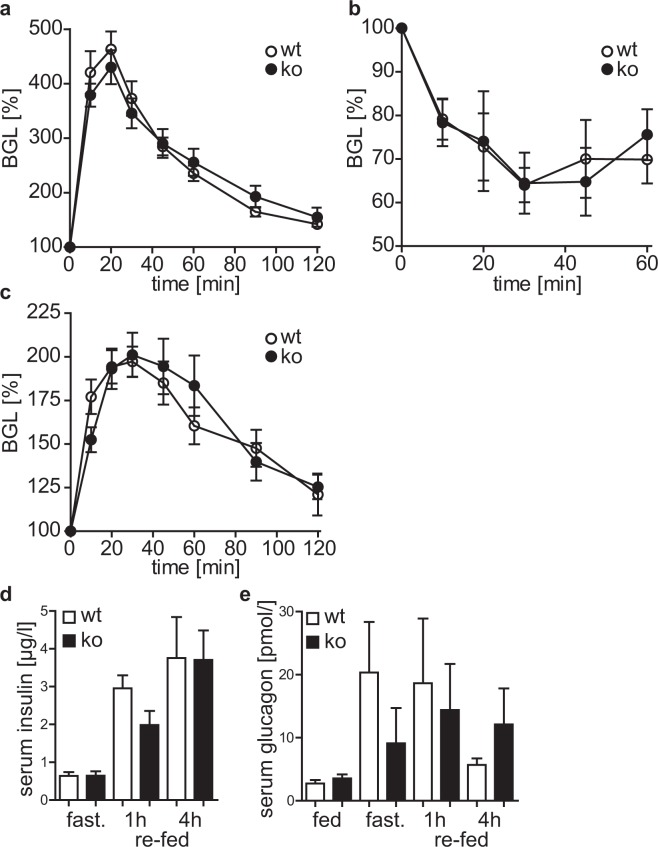
Cyth3^−/−^ mice are glucose tolerant and insulin tolerant. (**a**) Male and female wt (white circles) and cyth3^−/−^ (ko, black circles) mice were fasted overnight before i.p. glucose injection (2 mg/g of body weight). Blood glucose was measured every 10 to 30 minutes and calculated as % in comparison to the blood glucose levels (BGL) before glucose injection (n = 5–6, 2 male and 3–4 female mice). (**b**) Non-fasted male and female wt (white circles) and cyth3^−/−^ (ko, black circles) mice were injected i.p. with insulin (0.75U/kg of body weight), blood glucose was measured for one hour every 10 to 15 minutes (n = 5–6, 4–5 male and 1 female mice) and calculated as % in comparison to the BGL before insulin injection. (**c**) Male and female wt (white circles) and cyth3^−/−^ (ko, black circles) mice were fasted overnight before i.p. sodium pyruvate injection (2 mg/g of body weight). Blood glucose was measured every 10 to 30 minutes and calculated as % in comparison to the BGL before sodium pyruvate injection (n = 10, 7 male and 3 female mice). Serum levels of insulin (**d**) and glucagon (**e**) were analyzed of starved and subsequently re-fed male and female wt (white bars) and cyth3^−/−^ (ko, black bars) mice by ELISA (n ≥ 5, 2–4 male and 3–4 female mice). Results are given in means ± SEM.

The diminished IR-signaling in liver and WATi (Figs 1 and 2) prompted us to ask whether this leads to a glucose and insulin resistance of cyth3^−/−^ mice. Neither the clearance of blood glucose in a glucose and pyruvate tolerance test, nor the decrease of blood glucose after insulin injection was differentially regulated by the cyth3^−/−^ mice in comparison to wt mice (Fig. 3a–c). These findings indicate that cyth3, although important for IR-downstream signaling, is not required for glucose uptake and insulin-dependent regulation of gluconeogenesis. The BGL is balanced by the secretion of insulin and glucagon from the pancreas. Under all tested conditions, serum levels of both hormones were not significantly different between wt and cyth3^−/−^ mice (Fig. 3d,e), indicating that cytohesin-3 function is dispensable in the pancreas.

### Aged cytohesin-3 deficient mice have a reduced body weight, visceral fat, and blood glucose levels

**Figure 4:**
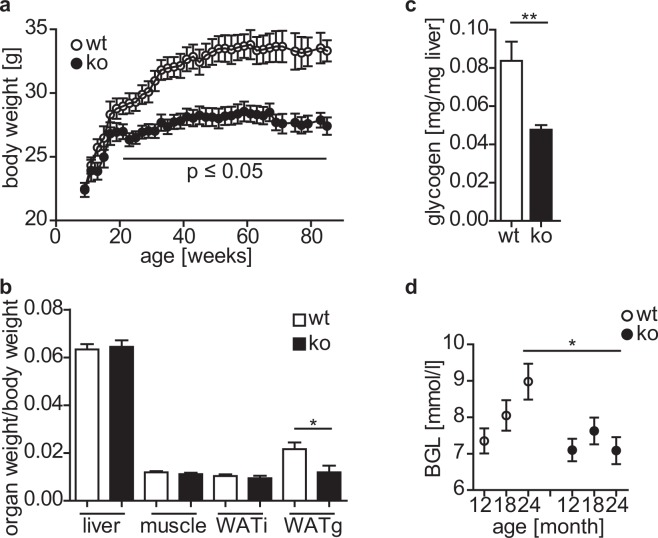
Cytohesin-3 deficiency leads to a reduction in body weight, visceral fat and BGL during aging. Body weight (**a**) of male mice (n ≥ 6) was monitored over two years. Organ weights were normalized to body weight from two years old male mice (n = 6) (**b**). Liver glycogen (n = 5) was analyzed from two years old male mice (**c**). Blood glucose levels (BGL) were monitored over two years (n ≥ 6) (**d**). The results are given in means ± SEM (*p < 0.05; **p < 0.01).

Aging is a major risk factor for the development of diabetes probably caused by changes in body composition (increase in body fat) and decreased physical fitness (reduced muscle mass)^10^. Since young adult cyth3^−/−^ mice did not show signs of type 2 diabetes, we analyzed the phenotype of two year old wt and cyth3^−/−^ mice (Fig. 4). In general, aged cyth3^−/−^ mice were leaner than wt mice (Fig. 4a). After 21 weeks, the difference became statistically significant. Two year old wt mice exhibited a significant age-related increase in body fat (WATi + 28%, WATg + 102%) and skeletal muscle mass was reduced by 16% (Supplementary Fig. S3a). In contrast, aged cyth3^−/−^ mice showed no significant change in WAT mass (WATi + 6% and WATG + 19%). The ratio of other organ weights to body weight was unaffected by the knockout (Fig. 4b, absolute organ weights are given in Supplementary Fig. S3b).

Reduced body weight and/or fat pad mass are also described for fat-specific IR-knockout mice^11,12^ and therefore point to an impairment of IR-signaling in cyth3^−/−^ mice. Furthermore, we found a 50% lower liver glycogen in knockout animals in comparison to wt mice (Fig. 4c). Expression of *Pnpla2*, an insulin dependent triglyceride lipase in adipose tissue, was significantly increased in WATg of aged cyth3^−/−^ mice (Supplementary Fig. S3c) indicating reduced insulin sensitivity. Serum levels of insulin, glucagon, triglycerides, and leptin were not affected by the cyth3-knockout (Supplementary Fig. S3d–g). Surprisingly, cyth3^−/−^ mice did not show the typical age-related increase of BGL as observed in wt mice (Fig. 4d).

These results show that cyth3^−/−^ does not lead to the development of type 2 diabetes, either in young or in aged animals, although several parameters in aged cyth3^−/−^ mice point to a disturbed IR-signaling and a strong effect of cyth3 on weight regulation.

### Under high-fat diet cytohesin-3-deficient mice exhibit reduced weight gain, body fat, and blood glucose levels

**Figure 5:**
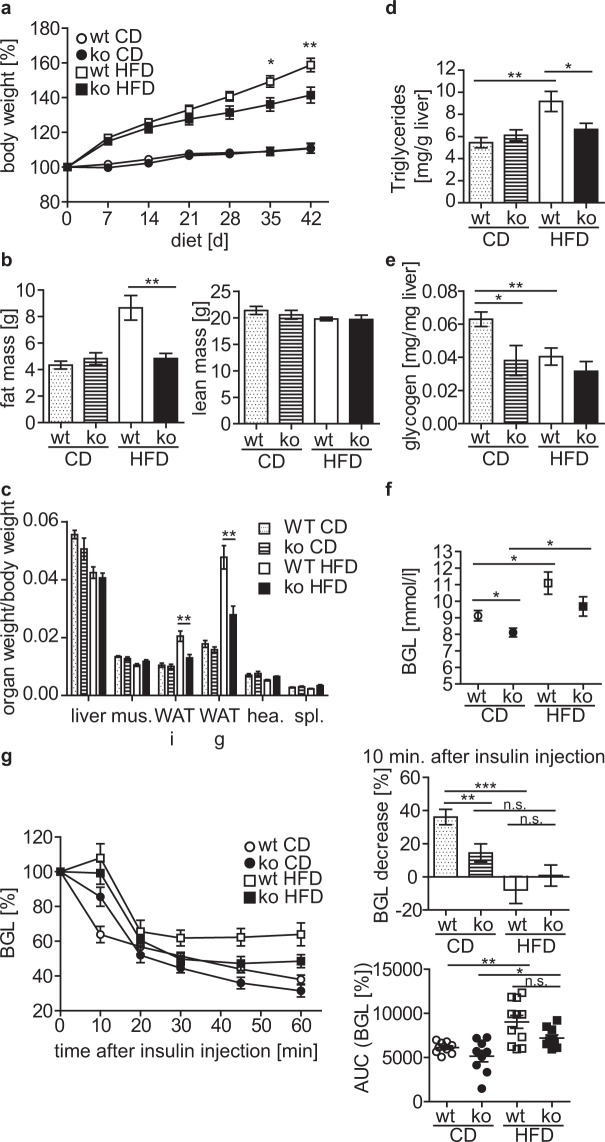
Cyth3^−/−^ mice show reduced weight gain during high-fat diet and develop an insulin resistance. Male wt (white symbols; white and dotted bars) and cyth3^−/−^ mice (ko, black symbols; hatched and black bars) received a high-fat diet (HFD, 60 kJ% fat) and a control diet (CD, 13 kJ% fat), respectively for 6 weeks. Weight gain was monitored over 6 weeks of diet (n ≥ 9) (**a**). Fat mass and lean mass determined by NMR (n = 7–8) (**b**), organ weights normalized to body weight (n = 8–11) (**c**), liver triglycerides, (**d**) liver glycogen (n = 8–11) (**e**) and blood glucose levels (BGL; n = 9–11) (**f**) were measured after 6 weeks of diet. (**g**) Male wt and cyth3^−/−^ (ko) mice were injected i.p. with insulin (0.75U/kg of body weight), blood glucose was measured for one hour every 10 to 15 minutes (n = 8–11) and calculated as % in comparison to the BGL before insulin injection. The bar diagram gives the decrease of the BGL 10 minutes after insulin injection (wt = white and dotted bars; (ko = hatched and black bars). Area under curve was calculated and depicted for every single mouse. Results are given in means ± SEM (*p < 0.05; **p < 0.01; ***p < 0.001, n.s. = not significant).

Since cyth3^−/−^ mice did not show the typical age-related increase in body fat as seen in wt mice, we asked whether we could phenocopy aged cyth3^−/−^ mice by using young mice on a high-fat diet (HFD). We fed mice a HFD (60 kJ% fat) and a normal-fat control diet (CD, 13 kJ% fat), respectively, for 6 weeks and again analyzed body and organ weight and determined liver glycogen and BGL (Fig. 5).

Body weight under CD conditions was comparable between wt and cyth3^−/−^ mice. Receiving a HFD led to a significant weight gain compared to mice fed with CD. Cyth3^−/−^ mice, however, showed significantly less weight gain on HFD compared to wt mice (Fig. 5a). Analysis of body composition using nuclear magnetic resonance showed a significantly higher fat mass in HFD-fed wt mice in comparison to HFD-fed cyth3^−/−^ mice, while the lean mass was similar (Fig. 5b). Comparable to aged cyth3^−/−^ mice, HFD-fed cyth3^−/−^ mice exhibited reduced weight of WAT depots while all other organs analyzed were normal in weight (Fig. 5c, Supplementary Fig. S4a,b). An increase in liver lipid content was not detected in cyth3^−/−^ mice in contrast to wt mice receiving HFD (Fig. 5d). Furthermore, similarly to aged mice, liver glycogen was significantly reduced in cyth3^−/−^ mice under CD compared to wt mice. While in wt mice, a HFD led to a significant reduction of liver glycogen, the already low amount of liver glycogen in CD-fed cyth3^−/−^ mice was not significantly reduced after HFD (Fig. 5e). The BGL increased significantly in both genotypes due to HFD compared to CD, but cyth3^−/−^ mice had a 11% (CD) and 13% (HFD) lower BGL compared to their wt counterparts (Fig. 5f). On one hand, this was consistently observed in young and aged cyth3^−/−^ mice with normal chow. On the other hand, this is in contrast to a former study which postulated a diminished insulin signaling accompanied by an increased BGL after inhibiting all cytohesin-proteins with secinH3 in mice^8^. To analyze if the cyth3-knockout mice have reduced insulin signaling under HFD and CD conditions, we performed an insulin tolerance test (Fig. 5g). Six weeks of HFD induced a significant insulin resistance in wt and cyth3^−/−^ mice, although HFD-fed cyth3^−/−^ mice were significantly lighter than their wt counterparts. In CD-fed cyth3^−/−^ mice, glucose clearance from the blood was significantly diminished 10 minutes after insulin administration compared to CD-fed wt mice, also indicating insulin-resistance. Therefore, we conclude that cyth3^−/−^ mice are insulin resistant independently of overweight and that the homeostatic BGL is differently regulated by cyth3 in contrast to the acute insulin stimulated glucose uptake.

### Cytohesin-3 deficient mice have an increased lipid excretion

**Figure 6:**
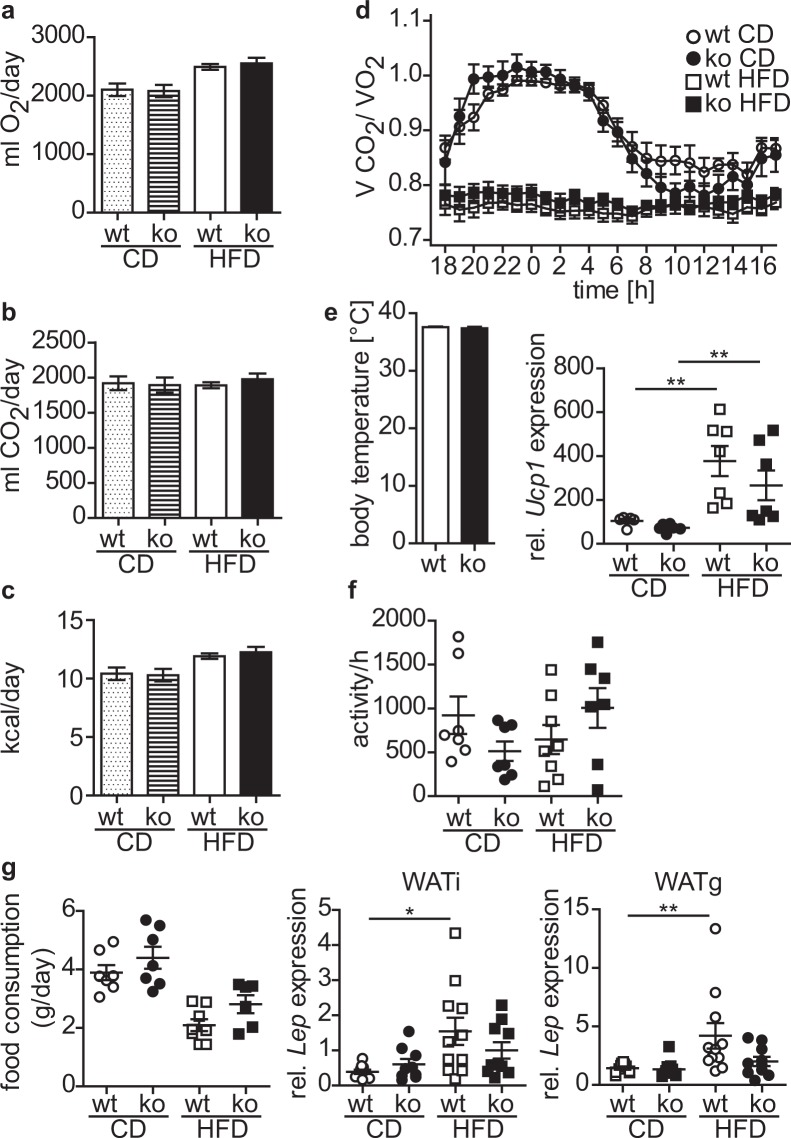
HFD-fed cytohesin-3 deficient mice have a normal metabolic rate. Male wt (white symbols; white and dotted bars) and cyth3^−/−^ mice (black symbols; hatched and black bars) received a high-fat diet (HFD, 60 kJ% fat) and control diet (CD, 13 kJ% fat), respectively. After 6 weeks mice were analyzed in metabolic cages for oxygen consumption (n = 7–8) (**a**) and carbon dioxide production (n = 7–8) (**b**). From these values energy expenditure (**c**) and the respiratory exchange ratio (**d**) were calculated. Body temperature was determined from HFD-fed mice (n = 4) (**e**) and *Ucp1* in brown adipose tissue was analyzed by PCR. Activity (n = 7–8) (**f**) and food intake (n = 6–8) (**g**) were also determined in metabolic cages. Gene expression of *Leptin* in WATi and WATg was analyzed by PCR. The results are given in means or as individual data points ± SEM (*p < 0.05; **p < 0.01).

We asked next whether the decreased weight gain of cyth3^−/−^ mice during a HFD is due to an increased metabolic rate. For this purpose we analyzed the energy expenditure of wt and cyth3^−/−^ mice in metabolic cages 6 weeks after receiving a CD and HFD, respectively. Oxygen consumption (Fig. 6a) and CO_2_ production (Fig. 6b) were similar between wt and cyth3^−/−^ mice, as was the energy expenditure (Fig. 6c). The respiratory exchange ratio was different between HFD and CD but independent of the genotype (Fig. 6d). Body temperature of wt and cyth3^−/−^ mice receiving a HFD was similar, which was supported by the comparable *Ucp1* expression in brown adipose tissue (Fig. 6e). The locomotor activity was overall unaltered by genotype or diet (Fig. 6f).

**Figure 7:**
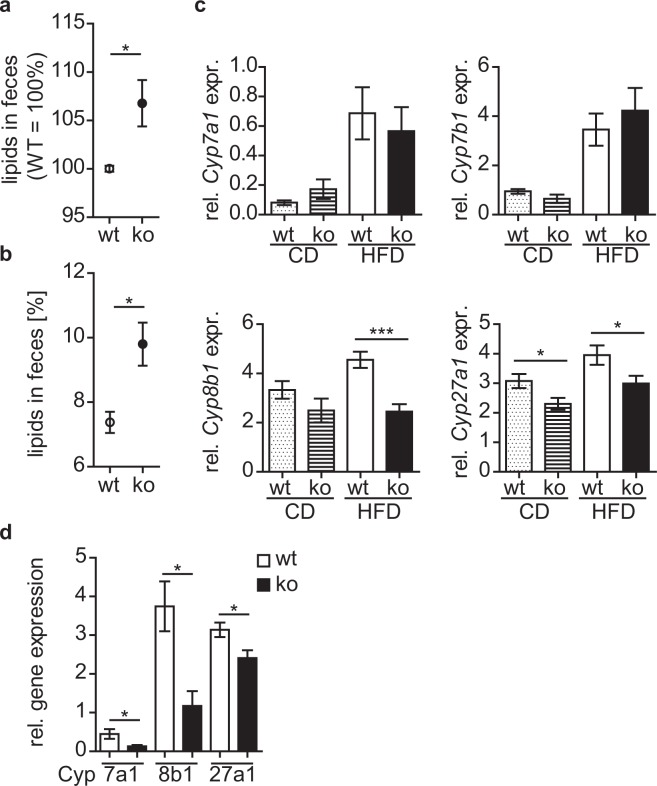
Cytohesin-3 deficient mice have an increased lipid excretion. Lipid excretion from wt (white symbols) and ko mice (black symbols) after HFD (n = 6–7) (**a**) and aged (in average 24 weeks old) wt and ko mice (wt, n = 3; ko, n = 5) (**b**) was analyzed by extraction from feces. Gene expression of *Cyp7a1*, *Cyp7b1*, *Cyp8b1* and *Cyp27a1* in livers from HFD- and CD-fed male wt (dotted and white bars) and ko mice (hatched and black bars) (Cyp7b1, n = 6–7; for all other Cyps n = 9–11) (**c**) and from two years old wt and cyth3^−/−^ male mice (n = 6) (**d**) was analyzed by PCR. The expression was normalized to *Hprt*. The results are given in means ± SEM (*p < 0.05; ***p < 0.001).

Since energy expenditure was similar, we asked whether cyth3^−/−^ mice are leaner due to a decreased food intake. The uptake of HFD was reduced compared to CD but was independent of the genotype (Fig. 6g). This finding was confirmed by *leptin* expression levels in WATi and WATg. Finally, we measured the lipid content in feces of wt and cyth3^−/−^ mice receiving a HFD. Lipid excretion was significantly enhanced in cyth3^−/−^ mice compared to wt, which was in line with the reduced body fat content (Fig. 7a). Likewise, we also found a significantly increased lipid excretion in aged cyth3^−/−^ mice compared to wt mice (Fig. 7b). An excess of lipid excretion can be the result of impaired digestion or absorption of fat. It was previously described that a liver-specific IR-knockout leads to a decreased expression of bile acid synthetic enzymes resulting in the formation of gallstones^13^. Although we could not observe gallstone formation in aged cyth3^−/−^ mice, we found a significantly lower expression of bile acid synthetic enzymes of the classical pathway, namely Cyp8b1 and Cyp27a1, in HFD-fed and aged cyth3^−/−^ mice compared to wt mice (Fig. 7c,d).

Taken together, reduced age-related and HFD-induced obesity in cyth3^−/−^ mice is not due to increased metabolic rates but most likely caused by an increased lipid excretion induced by a decreased bile acid synthesis.

## Discussion

Metabolic diseases like type 2 diabetes are widespread in western countries and depend on the combination of several risk factors like genetic causes, lifestyle (e.g. overweight), and age. Therefore, deciphering all participants in the regulation of insulin receptor (IR)-signaling is important for future treatments but also for the reduction of risk factors. In this paper, we describe for the first time the *in vivo* requirement of cyth3 for insulin signal transduction in liver and fat, as well as in body weight control in the mouse.

Glucose homeostasis is regulated by liver via glycolysis and gluconeogenesis and by adipose tissue mainly via secretion of adipokines^14^. While complete loss of insulin signaling in the liver in young mice leads to severe insulin resistance and glucose intolerance, aged liver-specific IR-deficient mice are glucose-tolerant^15^. This effect was reversed by reintroduction of IR-A, showing that also the expressed isoform of the IR is important for the mediated effects in young and aged animals^16^. Young fat-specific IR-deficient mice show a normal response to glucose and insulin injection but are protected from age-related glucose intolerance and insulin resistance^11^. In another study young and aged adipocyte-specific IR-knockout mice exhibited a reduced insulin responsiveness^12^. Impaired IR-signaling can be adverse, beneficial, or of no impact depending on the age and the affected tissue. Nevertheless, the mechanisms underlying these phenomena are still elusive.

Previous studies suggested a role for cytohesins in modulating IR-signaling^2,3,8^, e.g. inhibiting all cytohesins with secinH3 led to reduced hepatic insulin signaling in mice^2^. In this study, we identified an essential genetic role of cyth3 *in vivo* in IR-signaling in the liver. Regulation of *Gck* and *Pepck* expression, and activation of both branches downstream of the IR (i.e. the AKT-pathway and the ERK-pathway), were substantially disturbed while phosphorylation of the IR was not affected. Therefore, we conclude that cyth3 is important for full activation of IR-signaling in the liver upstream of AKT and ERK. Our *in vitro* studies in HepG2 cells point to a reduced IR-internalization in the absence of cyth3 and suggest this as potential mechanism which has been described to be important for the signaling capacity of the IR^17,18^.

The *in vivo* role of cyth3 in adipose tissue is unknown, although it has been shown that in the fibroblast-like adipocyte cell line 3T3-L1, cyth-2 and -3 are translocated to the plasma membrane after stimulation with insulin^19,20^ and that cyth3 is required for Glut4 translocation^7^. We now show that cyth3 is essential for IR-signaling in adipose tissue. The failure to up-regulate genes coding for *de novo* lipogenesis in cyth3^−/−^ mice was also reflected by a diminished generation of triglycerides and establishes cyth3 as a so far unknown regulator of lipid metabolism.

Aged cyth3^−/−^ mice have a reduced body weight, a reduced fat mass, and a lower BGL. The decreased body weight was not due to reduced body size, as determined by tibia length of young adult mice (Supplementary Fig. S5a). A reduced body weight, BGL, and liver glycogen as observed in aged cyth3^−/−^ mice, compared to wt mice, may also result from calorie restriction^21^. However, this is unlikely since weight gain after a period of starvation and daily nutrient uptake in young mice (Supplementary Fig. S5b,c), as well as food intake under CD and HFD conditions, was similar between wt and cyth3^−/−^ mice. Furthermore, caloric restriction leads to an overall weight loss measurable in total body weight and in all tissues^21^. In aged cyth3^−/−^ mice, we found a disproportionally reduced portion of WATg. This is remarkable since increased WATg is associated with metabolic dysfunction, while WATi has been shown to be protective^22–24^. Adipocyte-specific IR-knockout mice also have a reduced adipose tissue weight,^12^ as do other knockout mice with deletions in IR downstream signaling components like IRS1^25^ and S6Kinase 1^26^.

We challenged young mice with a HFD, which phenocopied aged cyth3^−/−^ mice which also exhibited a decreased body weight and body fat content. Resistance to HFD-induced obesity, despite normal energy expenditure and locomotor activity, could be explained by a reduced gastrointestinal fat absorption as we found an increased lipid excretion in aged and HFD-fed cyth3^−/−^ mice accompanied by a decreased expression of enzymes for bile acid synthesis. Knockout mice for bile acid transporters (e.g. Ostα^27^) or secretin-receptor, which regulates the fatty acid transporter CD36^28^, show a similar phenotype. On one hand, increased lipid excretion can be considered beneficial in controlling body fat accumulation. On the other hand, steatorrhea can be a sign of severe diseases, like pancreatic exocrine insufficiency, which can be caused by diabetes^29^. Although cyth3-deficient mice have reduced insulin signaling, they showed no symptoms of a severe pancreatic disease.

In the case of cyth3-deficiency, the reduced body and fat weight (as can be seen in aged and HFD-fed mice) can be a result of reduced IR-signaling in adipose tissue^11,12^ or reduced bile acid synthesis due to reduced IR-signaling in the liver^13^. At this stage, however, we cannot fully exclude that cyth3 regulates bile acid synthesis directly.

With this study, we presented the essential role of cyth3 *in vivo* in IR-downstream signaling in liver and fat that leads to the prevention of age- and diet-induced obesity and therefore suggest cyth3 as a potentially interesting novel therapeutic target for metabolic diseases.

## Methods

### Mice

Cyth3^−/−^ mice (Cyth3^tm2a(KOMP)Wtsi^) were obtained from KOMP Repository (UC Davis, USA). Male and female C57BL/6 N wild-type (wt) and cyth3^−/−^ mice originate from heterozygous breeding pairs and were bred under specific pathogen–free conditions at the animal facility of the University of Bonn. Littermates were used at 7–12 weeks and two years of age, respectively. All experiments for animal models were performed two to three times, and data were jointly analyzed. Animal care and experiments were performed according to German and Institutional guidelines for animal experimentation and were approved by the government of North Rhine-Westphalia (Germany).

### High-fat diet experiment

At the age of 6–8 weeks wt and cyth3^−/−^ mice received a high-fat diet (EF D12492 (I) mod. 60 kJ% fat (Lard) from Sniff, Soest, Germany) and a control diet (EF D12450B*mod. LS 13 kJ% fat from Sniff) for 6 weeks, respectively. Body weights during treatment were recorded once a week.

### Indirect calorimetry and body composition

Oxygen consumption and CO_2_ production were measured using a TSE Phenomaster indirect calorimetry system for 24 h. Animals were recorded in their home cages after habituation prior to the measurement period. Mice were kept at an ambient temperature of 23 °C with a 12 h dark-light cycle with free access to food and water. The measurement intervals were two minutes per cages. Energy expenditure was calculated using the Weir equation. Body composition (lean mass and fat mass) was quantified using a Bruker Minispec-LF TD-NMR device.

### Lipid extraction from feces

Lipid extraction from feces was performed as described in^30^. Briefly, feces was collected, weighted and homogenized in 5 ml of normal saline. After addition of 5 ml chloroform: methanol mix (2:1) the suspension was centrifuged at 1,000 × g. The lower liquid phase was collected in a fresh tube and dried for 3–4 days until all liquid was evaporated. Lipid mass was weighted and calculated as percentage of feces weight.

### Glucose tolerance pyruvate tolerance and insulin tolerance test

For the glucose tolerance test and pyruvate tolerance test, mice were fasted for 12 h and subsequently injected intraperitoneally (i.p.) with glucose (2 mg/g of body weight) and sodium pyruvat (2 mg/g of body weight), respectively. For the insulin tolerance test non-fasted mice were injected i.p. with human insulin (0,75 U/kg of body weight). Blood samples were taken from the tail tip every 10–15 minutes and blood glucose was determined with the Accu-chek system (Roche, Mannheim, Germany).

### ELISA

ELISAs were used for determination of insulin, glucagon (both mercodia, Uppsala, Sweden), leptin (Crystal Chem Inc., IL, USA) and triglycerides (Wako, VA, USA) according to manufacturer’s instructions.

### Measurement of liver-glycogen

Liver-glycogen was measured with the D-Glucose HK Assay Kit from Megazyme (Coring, Gernsheim, Germany). 10–20 mg liver was minced and cooked for one hour in 0.5 ml 2 M HCl and frequent vigorous shaking. Hydrolysis products were neutralized by adding 0.5 ml 2 M NaOH. Samples were centrifuged at 22000 × g for 10 minutes, 2–10 µl of supernatants were assayed.

### Reverse transcription and PCR

Total RNA was isolated with TRIzol® and transcribed into cDNA using the High-Capacity cDNA Reverse Transcription Kit (Applied Biosystems, CA, USA). Real-time PCR was performed on an icycler (Biorad, München, Germany) using the Kapa^TM^ SYBR Fast QPCR Mastermix (Peqlab, Erlangen, Germany). Expression levels were normalized to *Hprt* as housekeeping gene (MWG Operon, Cologne, Germany; primer sequences are listed in Table 1).

**Table 1:** Primer sequences.

gene	forward 5′-3′	reverse 5′-3′
cytochrome P450, family 7, subfamily a, polypeptide 1 (*Cyp7a1*)^13^	AGCAACTAAACAACCTGCCAGTACTA	CTGTCCGGATATTCAAGGATGCA
*Cyp7b1* ^13^	GAGGTTCTGAGGCTGTGCTC	TCCCTCCTTTGAAAAACGTGC
*Cyp8b1* ^13^	TGGCCTCTTTCACTTCTGCT	ATGGAAGAGACGCTGCAACT
*Cyp27a1* ^13^	GGAGGATTGCAGAACTGGAG	TGCGGGACACAGTCTTTACTT
Fatty acid synthase (*Fasn*)	TCTCGAGGAAGGCACTACAC	GTTGGACAGCAGGATACACC
Glucokinase (*Gck*)	GAACAACATCGTGGGACTTC	AGCTCCACATTCTGCATCTC
Glucosetransporter 4 *(Glut4)*	TCCTTTCTCATTGGCATCAT	GTTGGTTGAGTGTTCCCAAG
Hypoxanthine guanine phosphoribosyl transferase (*Hprt)*	GCTGGTGAAAAGGACCTCT	CACAGGACTAGAACACCTGC
Leptin (*Lep*)	TGGCTTTGGTCCTATCTGTC	TGGTCCATCTTGGACAAACT
Patatin-like phospholipase domain containing 2 (*Pnpla2*)	CTCCGAGAGATGTGCAAACA	CATGTTGGAAAGGGTGGTCA
phosphoenolpyruvate carboxykinase (*Pepck)*	TGTCGGAAGAGGACTTTGAG	CACTTGATGAACTCCCCATC
sterol regulatory element-binding protein 1c *(Srebp1c)*	GACCACAGAAAGGTGGAATC	CTACCTGGACTGAAGCTGGT
Uncoupling protein 1 *(Ucp-1)*	CTCCAGTGGATGTGGTAAAA	ACAATCCACTGTCTGTCTGG

### Western Blot analysis

Organs from wt and cyth3^−/−^ mice were homogenized on ice in MRC-lysis buffer (50 mM Tris-HCl, pH 7.5, 1 mM EGTA, 1 mM EDTA, 270 mM Sucrose, 1% Triton X-100, protease- and phosphatase-inhibitors). Western blots were performed as described previously^31^. Monoclonal rat anti-cytohesin-3 antibody was kindly provided by E. Krämmer (GMF, Munich, Germany), anti-pIR and secondary antibodies were received from Santa Cruz Biotechnology (TX, USA) all other primary antibodies were purchased from Cell Signaling (MA, USA).

### Culturing and insulin receptor internalization in HepG2 cells

HepG2 cells were cultured in RPMI containing 10% FCS, 100 U/mL penicillin and 0.1 mg/mL streptomycin. Cells were transfected with cytohesin-3 siRNA (target sequence: 5′GGAAUCAUCCCGUUGGAAA3′) or Renilla siRNA (both Dharmacon, Lafayette, CO, USA) as control with Lipofectamin® RNAiMAX. After 48 hours cells were starved for 24 h, stimulated with human insulin for 10 minutes and analyzed by western blot. For IR-internalization starved cells were stimulated with human insulin for 10–30 minutes in the presence of Brefeldin A. Surface expression of IR was determined by flow cytometry with a PE anti-human CD220 antibody (Biolegend, San Diego, CA, USA).

### Statistical analysis

Student’s *t* test (n ≥ 6 and Gaussian distribution) or Mann-Withney test (n ≤ 5 or no Gaussian distribution) were calculated with GraphPad Prism software (San Diego, CA, USA). Values of p < 0.05 were considered significant. Results are given as mean + SEM or in a scatter plot with individual data points.

## Supplementary information

Supplementary Dataset 1

## Data Availability

Data, materials and detailed protocols will be made available upon request.

## References

[1] Wild S, Roglic G, Green A, Sicree R, King H (2004). Global prevalence of diabetes: estimates for the year 2000 and projections for 2030. Diabetes care.

[2] Hafner M (2006). Inhibition of cytohesins by SecinH3 leads to hepatic insulin resistance. Nature.

[3] Lim J, Zhou M, Veenstra TD, Morrison DK (2010). The CNK1 scaffold binds cytohesins and promotes insulin pathway signaling. Genes & Development.

[4] Kolanus W (2007). Guanine nucleotide exchange factors of the cytohesin family and their roles in signal transduction. Immunol. Rev.

[5] Oatey PB (1999). Confocal imaging of the subcellular distribution of phosphatidylinositol 3,4,5-trisphosphate in insulin- and PDGF-stimulated 3T3-L1 adipocytes. The Biochemical journal.

[6] Clodi M (1998). Effects of general receptor for phosphoinositides 1 on insulin and insulin-like growth factor I-induced cytoskeletal rearrangement, glucose transporter-4 translocation, and deoxyribonucleic acid synthesis. Endocrinology.

[7] Li J (2012). Grp1 Plays a Key Role in Linking Insulin Signaling to Glut4 Recycling. Developmental Cell.

[8] Fuss B, Becker T, Zinke I, Hoch M (2006). The cytohesin Steppke is essential for insulin signalling in Drosophila. Nature.

[9] Gillingham AK, Munro S (2007). The Small G Proteins of the Arf Family and Their Regulators. Annu. Rev. Cell Dev. Biol.

[10] Wilson PW, Anderson KM, Kannel WB (1986). Epidemiology of diabetes mellitus in the elderly. The Framingham Study. The American journal of medicine.

[11] Bluher M (2002). Adipose tissue selective insulin receptor knockout protects against obesity and obesity-related glucose intolerance. Developmental cell.

[12] Friesen M, Hudak CS, Warren CR, Xia F, Cowan CA (2016). Adipocyte insulin receptor activity maintains adipose tissue mass and lifespan. Biochemical and biophysical research communications.

[13] Biddinger SB (2008). Hepatic insulin resistance directly promotes formation of cholesterol gallstones. Nature medicine.

[14] Rosen ED, Spiegelman BM (2006). Adipocytes as regulators of energy balance and glucose homeostasis. Nature.

[15] Michael MD (2000). Loss of insulin signaling in hepatocytes leads to severe insulin resistance and progressive hepatic dysfunction. Mol. Cell.

[16] Diaz-Castroverde S (2016). Insulin receptor isoform A ameliorates long-term glucose intolerance in diabetic mice. Disease models & mechanisms.

[17] Di Guglielmo GM (1998). Insulin receptor internalization and signalling. Molecular and cellular biochemistry.

[18] Posner BI (2017). Insulin Signalling: The Inside Story. Canadian journal of diabetes.

[19] Venkateswarlu K, Oatey PB, Tavaré JM, Cullen PJ (1998). Insulin-dependent translocation of ARNO to the plasma membrane of adipocytes requires phosphatidylinositol 3-kinase. Current Biology.

[20] DiNitto JP (2007). Structural Basis and Mechanism of Autoregulation in 3-Phosphoinositide-Dependent Grp1 Family Arf GTPase Exchange Factors. Molecular Cell.

[21] Wetter TJ (1999). Effect of calorie restriction on *in vivo* glucose metabolism by individual tissues in rats. American Journal of Physiology - Endocrinology and Metabolism.

[22] Gabriely I (2002). Removal of Visceral Fat Prevents Insulin Resistance and Glucose Intolerance of Aging: An Adipokine-Mediated Process?. Diabetes.

[23] Booth, A. Magnuson, A. & Foster, M. Detrimental and protective fat: body fat distribution and its relation to metabolic disease. *Hormone Molecular Biology and Clinical Investigation* **17** (2014). 10.1515/hmbci-2014-0009 25372727

[24] Pitombo C (2006). Amelioration of diet-induced diabetes mellitus by removal of visceral fat. Journal of Endocrinology.

[25] Selman C (2007). Evidence for lifespan extension and delayed age-related biomarkers in insulin receptor substrate 1 null mice. The FASEB Journal.

[26] Selman C (2009). Ribosomal protein S6 kinase 1 signaling regulates mammalian life span. Science (New York, N.Y.).

[27] Wheeler SG (2014). Ostα^−/−^ mice exhibit altered expression of intestinal lipid absorption genes, resistance to age-related weight gain, and modestly improved insulin sensitivity. American journal of physiology. Gastrointestinal and liver physiology.

[28] Sekar R, Chow BKC (2014). Secretin receptor-knockout mice are resistant to high-fat diet-induced obesity and exhibit impaired intestinal lipid absorption. FASEB journal: official publication of the Federation of American Societies for Experimental Biology.

[29] Talukdar R, Reddy DN (2017). Pancreatic Exocrine Insufficiency in Type 1 and 2 Diabetes: Therapeutic Implications. The Journal of the Association of Physicians of India.

[30] Kraus, D. Yang, Q. & Kahn, B. B. Lipid Extraction from Mouse Feces. *Bio Protoc*. e1375 (2015). 10.21769/bioprotoc.1375 PMC4838042 27110587

[31] Mitschka S (2015). Co-existence of intact stemness and priming of neural differentiation programs in mES cells lacking Trim71. Sci. Rep.

