# Supplementary material for: Cytohesin-3 is required for full insulin receptor signaling and controls body weight via lipid excretion
Source: Sci Rep. 2019 Mar 5;9:3442. doi: 10.1038/s41598-019-40231-3 (PMC6401384; doi:10.1038/s41598-019-40231-3)
Supplement: Supplementary file 1 — Supplementary Dataset 1 [file 41598_2019_40231_MOESM1_ESM.pdf]

Cytohesin-3 is required for full insulin receptor signaling and controls body weight via lipid excretion

Bettina Jux, Dominic Gosejacob, Felix Tolksdorf, Christa Mandel, Michael Rieck, Angrit Namislo, Alexander Pfeifer, Waldemar Kolanus

Supplementary Figure 1

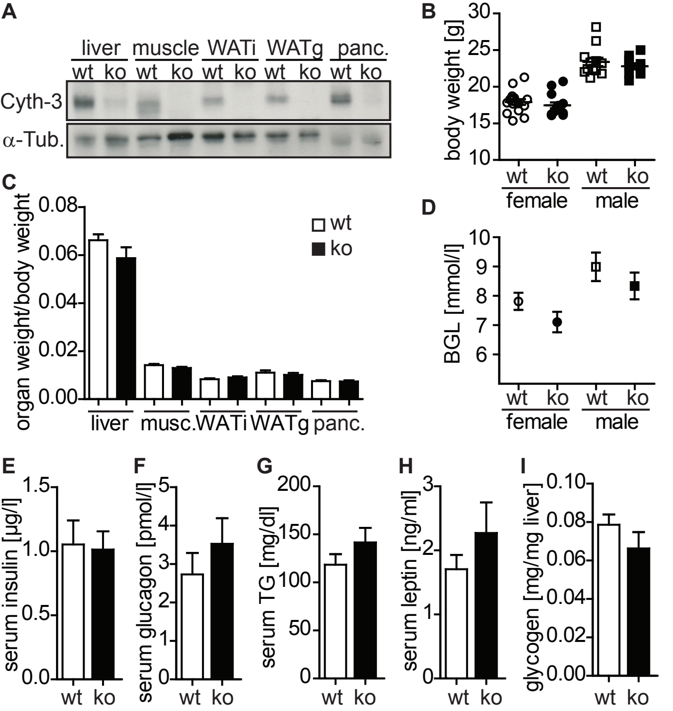

Suppl. Figure 1: Young adult cyth3$^{-/-}$ mice have a normal metabolic phenotype.

Male and female mice at the age of 7-12 weeks were phenotypically analyzed. A) A representative western blot analysis of the cytohesin-3 protein expression in liver, skeletal muscle, inguinal subcutaneous white adipose tissue (WATi), intra-abdominal gonadal white adipose tissue (WATg) and pancreas (panc.) is shown. Body weight at the age of 8-9 weeks (n ≥ 10) (B), organ weight normalized to body weight from male mice (n = 7-9) (C), blood glucose levels (BGL) in non-fasted mice at the age of 8-9 weeks (n ≥ 12) (D), serum levels of insulin (n = 8) (E), glucagon (n = 7) (F), triglycerides (TG; n = 5) (G), leptin (n = 6) (H) and liver glycogen (n = 6) (I) are given from male and female wt (white symbols and bars) and cyth3$^{-/-}$ (ko, black symbols and bars) mice as bar graphs of means + SEM or as individual data points ± SEM.

Supplementary Figure 2

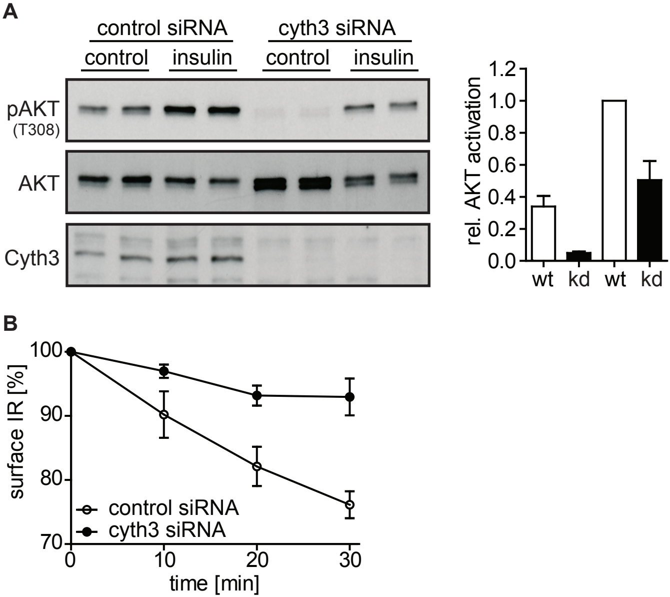

Supplementary Figure 2: IR signaling and internalization in HepG2 cells is impaired after cyth3-knockdown. **A**) Control siRNA (wt) or Cyth3 siRNA (kd) treated HepG2 cells were stimulated with 100nM insulin. Ten minutes later cells were harvested and activation of AKT was determined as a ratio of phosphorylated to total protein levels. Representative Western blot analyses are shown. The activation was normalized to insulin-stimulated wt cells (n = 4). **B**) IR internalization after indicated time points of insulin stimulation was measured by FACS and calculated in comparison to IR surface expression before stimulation (= 100%). Results are given in means ± SEM (n = 5).

Supplementary Figure 3

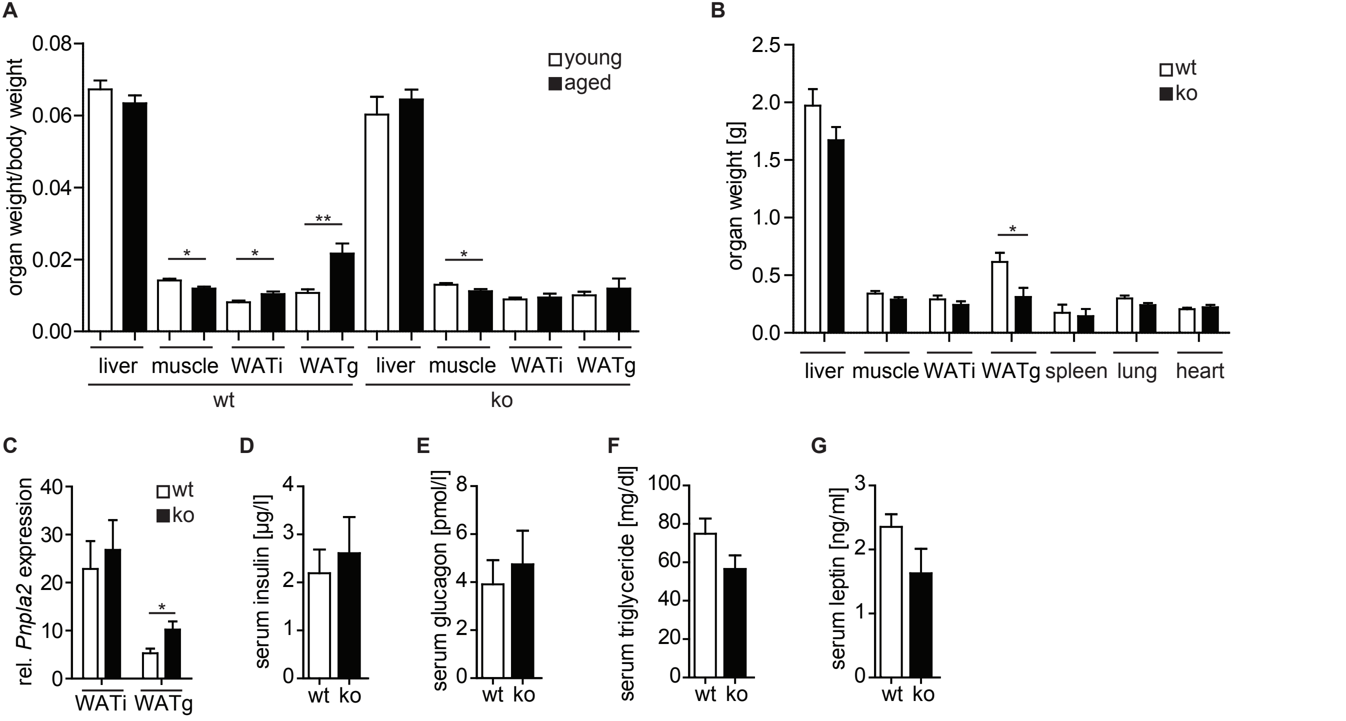

Supplementary Figure 3: Phenotypic analysis of two years old cyth3$^{+/+}$ (wt) and cyth3$^{-/-}$ (ko) mice. Comparison of organ weights relative to body weight from young (white bars) and aged mice (black bars) of wt and ko mice (n ≥ 6) (**A**). Total organ weights of aged wt (white bars) and ko mice (black bars) (n = 6) (**B**). Expression of Pnpla2 in adipose tissues was analyzed by PCR. The expression was normalized to Hprt (n = 5-6) (**C**). Serum levels of insulin (n = 6) (**D**), glucagon (n = 5) (**E**), triglycerides (n = 6) (**F**), and leptin (n = 6) (**G**) are given from aged wt (white bars) and ko (black bars) mice. Results are given as bar graphs of means + SEM (*, $p < 0.05$; **, $p < 0.01$).

Supplementary Figure 4

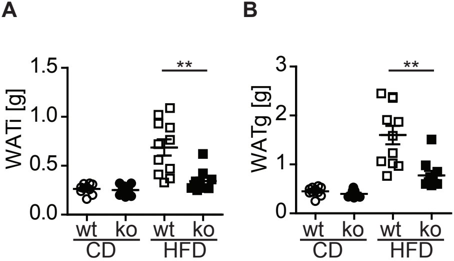

Supplementary Figure 4: Fad pad weight is reduced in HFD-fed cyth3$^{-/-}$ mice. Inguinal subcutaneous (WATi; **A**) and intra-abdominal gonadal white adipose tissue (WATg; **B**) from wt (white symbols) and ko mice (black symbols) was weight after 6 weeks of HFD (squares) and CD (cycles), respectively. Results are given as individual data points ± SEM (**, $p < 0.01$).

Supplementary Figure 5

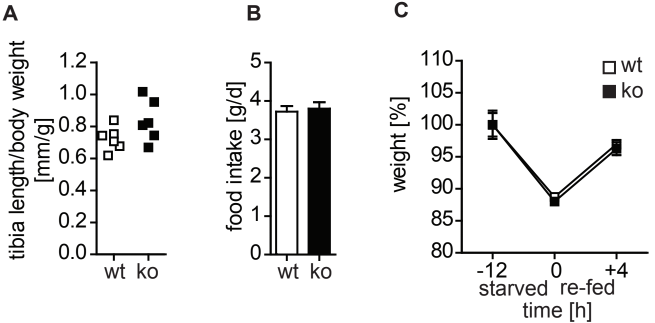

Supplementary Figure 5: Size and food uptake are similar between cyth3$^{+/+}$ (wt) and cyth3$^{-/-}$ (ko) mice. **A**) Mouse-size was determined by tibia lenght in relation to body weight of 8-9 weeks old mice (n = 6). **B**) Daily food intake of wt and cyth3$^{-/-}$ mice (n = 6). **C**) Weight loss and gain of wt (white squares) and cyth3$^{-/-}$ (ko, black squares) mice after a starvation period of 12 hours (-12) and subsequent re-feeding for four hours (+4) (n ≥ 12) Results are given in means ± SEM.

Full western blot images for Figure 1

liver pAKT^{T308}

wt ko wt ko

control insulin

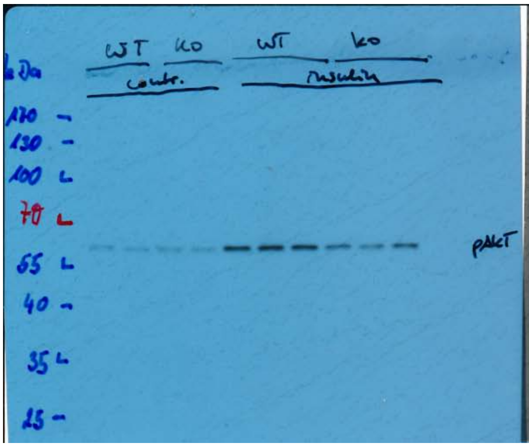

liver tAKT

wt ko wt ko

control insulin

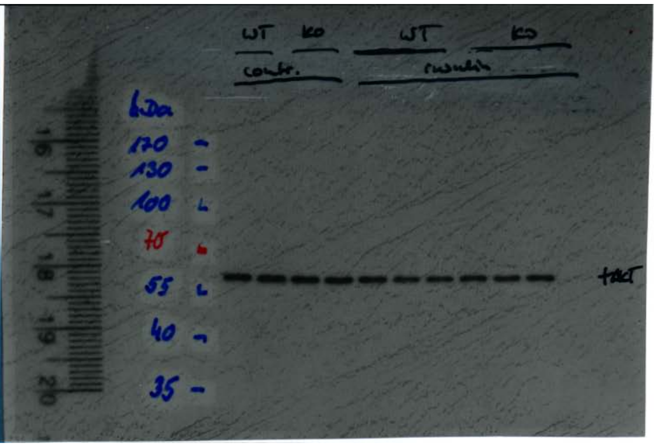

liver pERK$^{T202/204}$

wt ko wt ko

control insulin

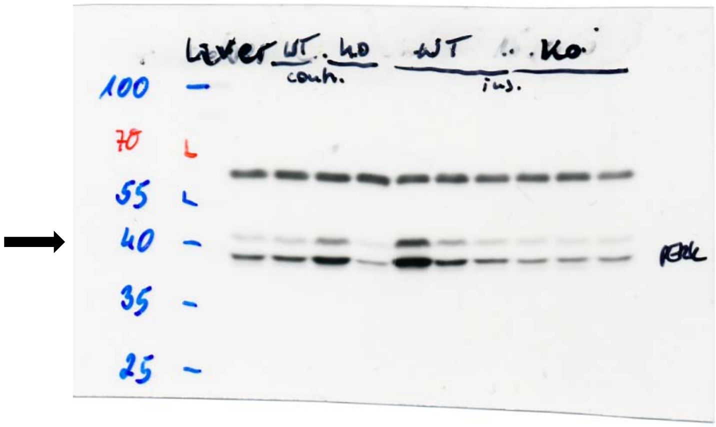

liver tERK

wt ko wt ko

control insulin

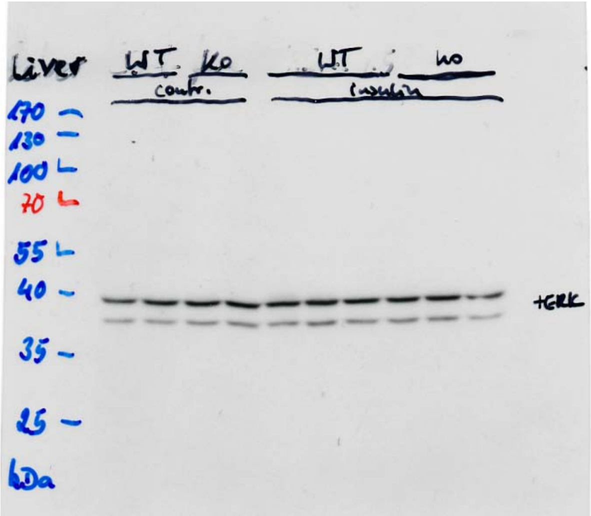

liver pIR$^{Y1162/3}$

wt ko wt ko

control insulin

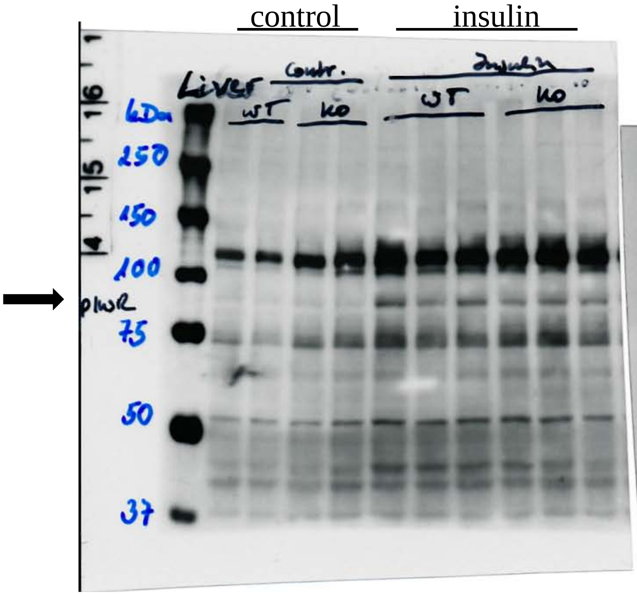

liver tIR

wt ko wt ko

control insulin

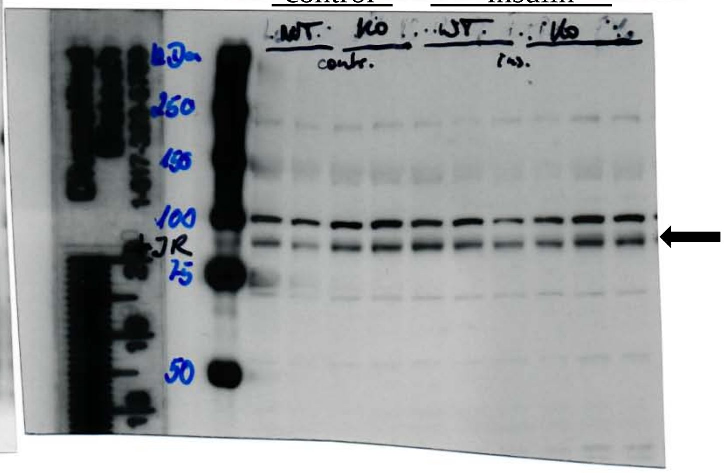

Full western blot images for Figure 2

WATi pAKT^{T308}

wt ko wt ko

control insulin

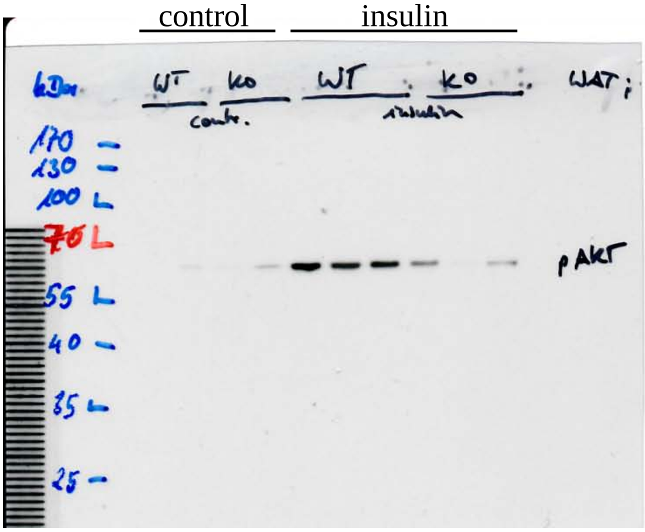

WATi tAKT

wt ko wt ko

control insulin

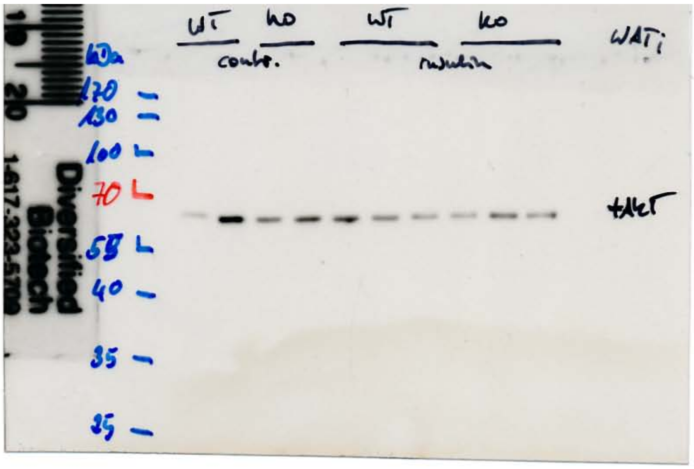

WATi pERK$^{T202/204}$

wt ko wt ko

control insulin

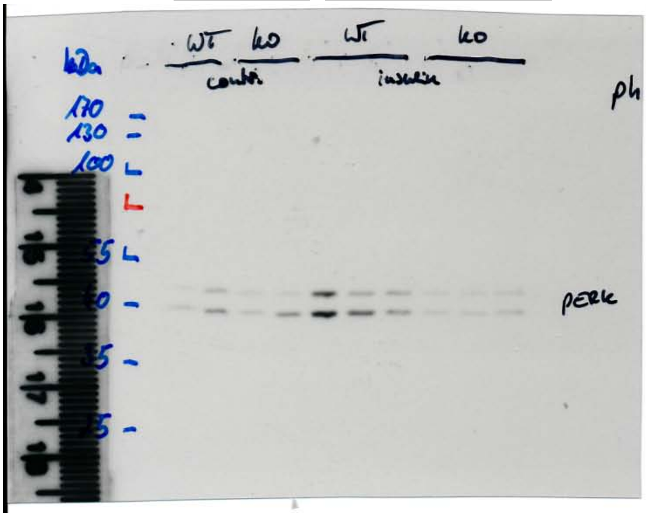

WATi tERK

wt ko wt ko

control insulin

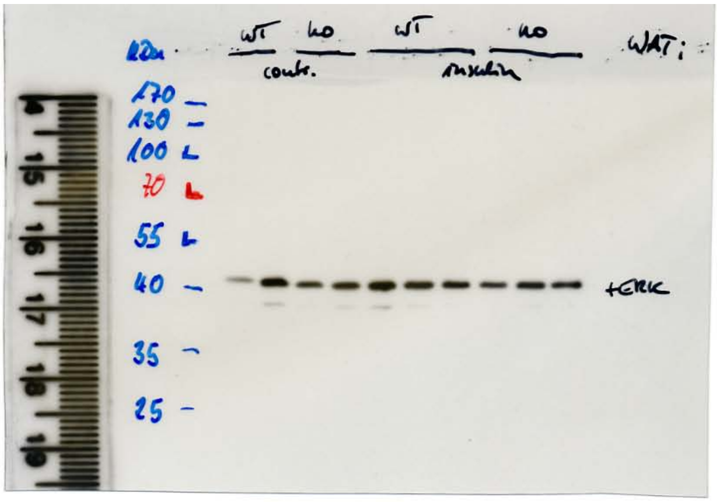

Full western blot images for Supplementary Figure 1

Cytohesin-3

liver musc. WATi WATg panc.

wt ko wt ko wt ko wt ko wt ko

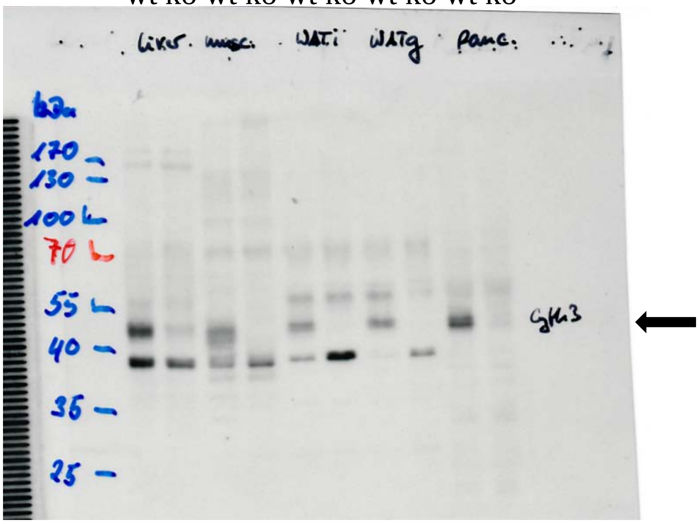

α-Tubulin

liver musc. WATi WATg panc.

wt ko wt ko wt ko wt ko wt ko

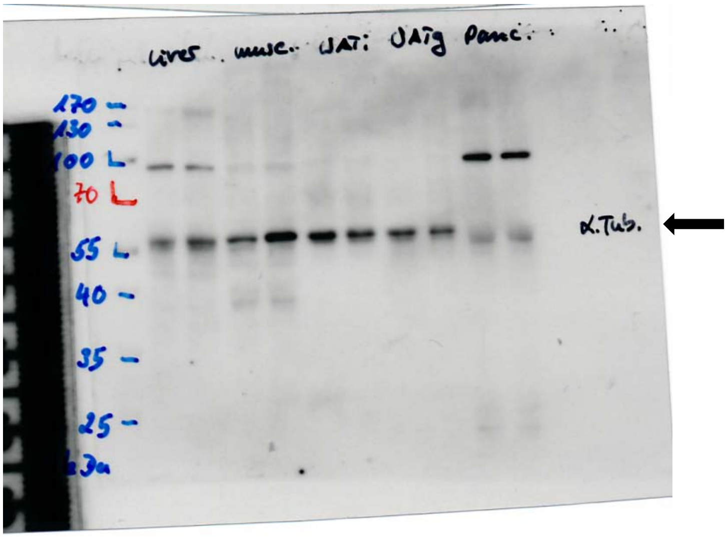

Full western blot images for Supplementary Figure 2

control siRNA   Cyth3 siRNA

contr. insulin   contr. insulin

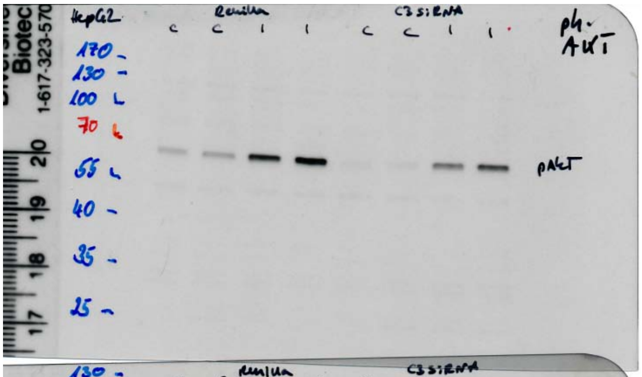

pAKT^{T308}

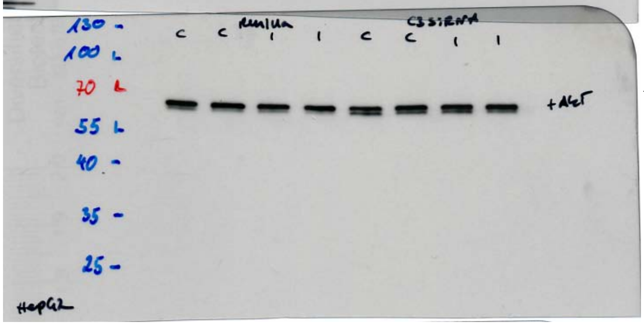

tAKT^{T308}

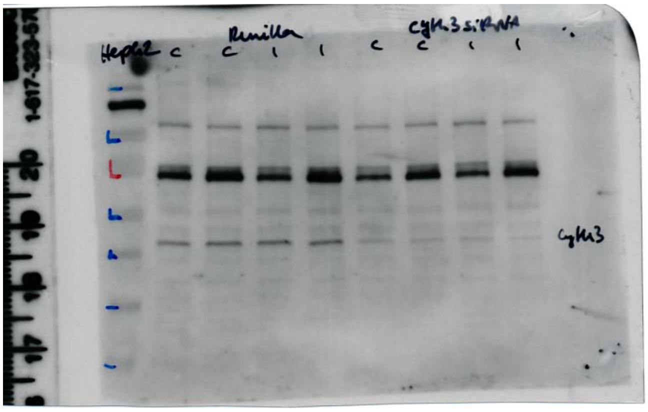

Cyth3
